# Estimation of the Structural and Geomechanical Anisotropy in Fault Gouges Using 3D Micro-Computed Tomography (μ-CT)

**DOI:** 10.3390/s20174706

**Published:** 2020-08-20

**Authors:** Eomzi Yang, Tae Sup Yun, Kwang Yeom Kim, Seong Woo Moon, Yong-Seok Seo

**Affiliations:** 1 School of Civil and Environmental Engineering, Yonsei University, Seoul 03722, Korea; did8810@yonsei.ac.kr; 2 Department of Energy & Resources Engineering, Korea Maritime & Ocean University, Busan 49112, Korea; kykim@kmou.ac.kr; 3 Department of Earth and Environmental Sciences, Chungbuk National University, Cheongju 28644, Korea; msw2080@cbu.ac.kr

**Keywords:** X-ray CT-based estimation, fault gouge, anisotropy of structure, geomechanical anisotropy

## Abstract

Fault gouges play an important role in the shear deformation of fault zones, by causing weakness and frictional instability in structures. Previous studies have investigated the evolution of shear deformation of fault zones by observing experiments using remolded and synthetic gouge specimens at a micro-scale. However, how the spatial configuration of the rock constituents accounts for the 3D anisotropy of intact structures of fault gouges, particularly at the core-scale, is not well understood. We obtained 3D μ-CT images of directionally cored gouge specimens and performed statistical analysis to quantify the major orientation of the internal structures. Direct shear tests were conducted to investigate the relationship between the distribution of the internal structures and geomechanical behavior. The results show that the undisturbed fault gouge has a clear anisotropy parallel to the fault plane even at the core-scale. Moreover, the direct shear test results show that the frictional resistance of a fault gouge has anisotropy related to the fault plane. The simple, yet robust method proposed in this study confirms that the core-scale structural anisotropy is correlated to the anisotropic shear resistance.

## 1. Introduction

It has been reported that the unique structure of a fault zone can degrade the geotechnical stability of the infrastructure or construction site; therefore, the evaluation of the current state of the fault zone is crucial in order to estimate the future risks related to the site [1,2]. Several efforts have been made to investigate the relationship between fault-slip data at the field scale and the stress state of a fault zone [3,4]. Moreover, the fault gouge, often found in the fault core, which accommodates most of the deformation in the fault zone has been widely studied because the anisotropy in a fault gouge and its origin provide clues for understanding the geological and deformative processes in the region [5]. Planar shear fabrics in shear bands are regarded as a fault-slip indicator and these have been examined at micro- to macro-scales to evaluate their functionality as a marker of shear displacements and their role during shear deformation [6,7,8,9]. Visual inspection using SEM images has also revealed that shear fabrics developed by the evolution of the strain state have planar anisotropy and their orientation is interpreted as foliation or discontinuity in fault rocks [10,11,12,13]. However, the frictional behaviors during shearing are governed by the spatial configuration of grains, clay matrix, discontinuity, cracks, and their interplay. In particular, as the laboratory studies on deformation are mostly conducted at the core-scale (e.g., ring shear and direct shear tests), evaluating the anisotropy of fault gouges at the core-scale is important for a better understanding of anisotropy-induced frictional behaviors.

The use of quantitative analysis for the characterization of the anisotropy in fault rocks has been investigated; Shape-preferred orientation (SPO), which represents the anisotropy in a fault rock via the distribution of principal axes of non-equant grains, suggests a relationship between the principal vector and shear zone boundary. A visual inspection of well-prepared, thin sections to collate the orientation of individual grains in three orthogonal planes has been implemented for the statistical analysis [14]. However, the applicability of SPO is often limited, owing to the time-consuming work related to the preparation of the sample and the possibility of out-of-plane components [15]. Anisotropy of magnetic susceptibility (AMS) has been widely used in research related to clay-rich gouges as a kinetic indicator of tectonic movement [16,17]. However, it has been reported that the spatial distribution of iron oxides and magnetite in gouges, which are the crucial carriers of magnetic susceptibility, may result in a biased estimation for the orientation of foliation [18,19]. X-ray texture goniometry (XTG) has also been used to examine the orientation of clay fabrics in natural gouges from different tectonic environments [20]. Further, 3D X-ray computed tomographic (CT) imaging has enabled the investigation of the internal constituents and estimation of the orientation of clasts in fault gouges [21,22].

Therefore, this study aimed to quantify the orientation of embedded features of intact fault cores and overcome the challenges of previous 2D analysis and qualitative methods by maintaining a coincident scale for the experiments and analysis. Contrary to previous quantitative analyses defining indicators that represent the anisotropic structure of fault gouge (e.g., SPO, AMS, XTG, etc.), this study attempted to evaluate the anisotropy induced from all of the constituents in the intact fault gouge by using slicing plane method (SPM) [23]. The reliability of SPM has been verified for characterizing the anisotropy in several types of fresh rocks, but it has not been verified for cataclastic rocks. Multiple undisturbed round-cut samples were recovered in the field and subjected to 3D X-ray CT imaging followed by the statistical estimation of anisotropy in the fault core. The effect of anisotropic structure on shear resistance was also investigated via direct shear tests along four different shearing directions, without remolding of the specimens.

## 2. Materials and Methods

### 2.1. Fault Gouge Specimens

**Figure 1:**
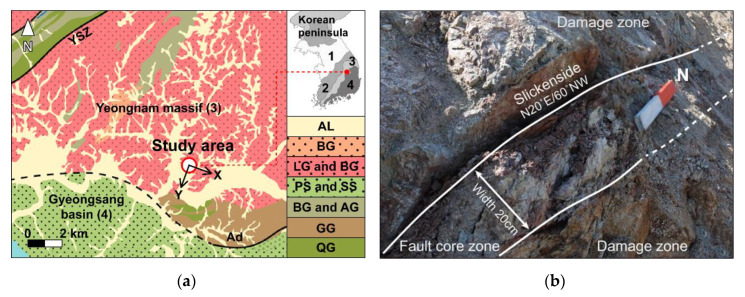
Field investigation located in 128.63° E/36.56° N. (**a**) Geological map of the investigation site (Alluvium: AL; Biotite Granite: BG; Leucocratic Granite and Biotite Granite: LG and BG; Purplish Siltstone and Sandstone: PS and SS; Banded Gneiss and Augen Gneiss: BG and AG; Granitic Gneiss: GG; Quartz-feldspar Gneiss: QG) The position of the Yeongnam Massif and the Gyeongsang Basin correspond to the numbers denoted in the map of the Korean Peninsula on the upper right. (1: Gyeonggi Massif; 2: Okcheon Belt; 3: Yeongnam Massif; 4: Gyeongsang Basin). The arrows stand for the coordinate system of this study. (**b**) A photograph of the outcrop taken during the field investigation.

**Figure 2:**
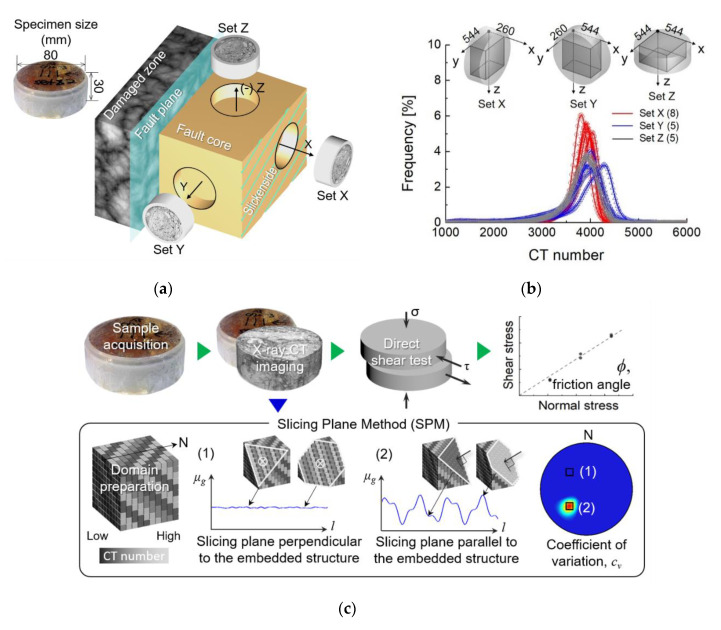
Sample preparation and experimental procedures. (**a**) Geometry of the fault zone and the coordinate system of X-ray CT images (**b**) Distribution of the CT numbers of each specimen (**c**) Experimental procedure (*l* is the distance of the slicing plane from the center of a domain).

The investigation site is located in Andong city, South Korea (128.63 °E and 36.56 °N) as shown in Figure 1. The major tectonic lines close to the investigation site run along both the Andong Fault (AF) and the Yecheon Shear Zone (YSZ). The AF, which trends E-W to E-N-E, is a boundary fault between the Gyeongsang Basin and the Precambrian-Jurrasic basement called the Yeongnam Massif [24,25]. While the part of the Gyeongsang Basin near the investigation site mainly consists of purplish siltstone and sandstone, the Yeongnam Massif is composed largely of leucocratic granite and biotitic granite, with a small portion of granitic gneiss near the AF. The dextral strike-slip YSZ runs along an E-N-E trend. The YSZ is regarded as a clue to understanding the crustal evolution in east Asia and the Korean Peninsula because of the similarity between the deformative aspect of YSZ and the tectonic movement of the East Asian continent [26,27,28,29,30]. The fault zone under investigation is within 10 km from the YSZ, and the fault plane is identified by its slickenside (N20° E/60° NW). The coordinate system (x- and *y*-axis) in this study was determined by the orientation of the fault plane as marked by the arrows. The fault core zone has a width of ~20 cm along the N20° E direction (Figure 1b). The round-cut samples were cored along three directions orthogonal to each other. The coordinate system in this study follows the geometry of the fault zone (Figure 2a); the *x*-axis is perpendicular to the fault plane, the *y*-axis is parallel to the strike of the fault plane, and the *z*-axis is normal to the xy-plane. A total of 24 cylindrically-shaped specimens were prepared with a height of 30 mm and a diameter of 80 mm: 12 in the x-direction (Set X), and 6 each in both the y- (Set Y) and z-direction (Set Z). Thorough plastic wrapping prevented the evaporation of internal moisture and helped maintain the in situ conditions.

### 2.2. μ-CT Image-Based Analysis

X-ray CT images were obtained for 18 undistributed specimens (8 for Set X, 5 for Set Y, and 5 for Set Z) using an industrial CT device (PCT-G3, SEC Ltd., Orlando, FL, USA). The image resolution was *dx* = 80 μm and the image set was stacked along the height to make a cylindrical 3D CT image stack. Since the coring directions varied, the 3D CT image stacks were rotated and resliced in the z-direction to create the domain with a size of approximately 260 × 544 × 544 voxels, as illustrated in Figure 2b. The CT number, also called the Hounsfield unit (HU), is represented by the X-ray attenuation coefficients shown in Equation (1).

(1) $$CT\;number\;(\mathrm{HU}\;\mathrm{scale})\;=\frac{\mu_{m}-\mu_{w}}{\mu_{w}}\times 1000$$

where $\mu_{m}$ and $\mu_{w}$ are the X-ray attenuation coefficients of the target material and water, respectively. The distribution of CT numbers for each directionally cored specimen ranges generally from 3500 HU to 4500 HU with a single modal shape, while the distribution of CT numbers for Set Y differs slightly from that of the other sets.

The CT number of a voxel is proportional to the relative density of materials constituting the voxel and the effective atomic number of those constituents; therefore, the strategic quantification of the spatial variability of the CT number naturally demonstrates how microstructural features are clustered. In our previous study [23], it was shown that the SPM can be used for the systematic scanning of clustered CT numbers in a domain and to quantify the 3D anisotropy in fresh rocks. Here we briefly describe the SPM procedure as follows (Figure 2c). The imaginary slicing plane defined at the center of the 3D CT image stack and the corresponding normal vector are determined. The group of the CT numbers “*g*” of the voxels on the slicing plane is collected and its mean value $\mu_{g}$ is computed. The slicing plane advances by unit distances along the given normal vector and the set of $\mu_{g}$ is successively obtained. Based on $\hat{\mu_{g}}$, the set of $\mu_{g}$, the coefficient of variation ($c_{v}=\sigma^{\mu_{g}}/\mu^{\mu_{g}}$) was obtained where $\sigma^{\mu_{g}}$ is the standard deviation of $\hat{\mu_{g}}$ and $\mu^{\mu_{g}}$ is the arithmetic average of $\hat{\mu_{g}}$. This process is repeated for all directions in 3D. If the domain has planar anisotropy and the slicing planes are perpendicular to the anisotropic orientation, shown as case “1”, the fluctuation of $\mu_{g}$ is not significant, while the slicing plane advances directly along the normal vector (Figure 2c). When the slicing plane is aligned parallel to the embedded anisotropy, shown as case “2”, as the slicing plane advances along the normal vector shown as a black arrow, $\mu_{g}$ noticeably fluctuates. Similarly, the highest $c_{v}$ is obtained when the slicing plane is aligned parallel to the embedded anisotropy; $c_{v}$ decreases as the slicing plane deviates from the orientation of the anisotropy. The map of $c_{v}$ values, plotted in stereonet using lower-hemisphere projection, presents the dominant orientations of the major anisotropic structures.

### 2.3. Direct Shear Test

In this study, the experiment was performed in two stages (Figure 2c). After the X-ray CT images of the intact fault gouge were taken, we experimentally measured the shear strength of the core specimens along four different shearing directions; τ_xy_, τ_xz_, τ_yz_, and τ_zy_. The first subscript of τ indicates a shearing plane normal to the axis, and the second subscript stands for the shearing direction. Thus, both τ_xy_ and τ_xz_ represent the shear resistance parallel to the fault plane. τ_yz_ is measured perpendicular to both the fault plane and the fault strike while τ_zy_ is a measure of the shear resistance perpendicular to the fault plane but parallel to the fault strike. Direct shear tests were conducted for six specimens along each direction immediately after the removal of the plastic wrap and specimen preparation. The strain rate was controlled at 1 mm/min (ASTM D 3080) until the shear displacement reached 15% of the specimen diameter. The normal stresses of 54 kPa, 107.9 kPa, and 161.9 kPa were applied to each shearing direction. The load cell measured the horizontal force per every 1 s until the specimen failed and the stress-strain curves were obtained. For each shearing direction, the friction angle was determined from the tangential slope based on Mohr–Coulomb’s criteria.

## 3. Results and Discussion

### 3.1. Anisotropy in Fault Gouges (μ-CT Imaging)

**Figure 3:**
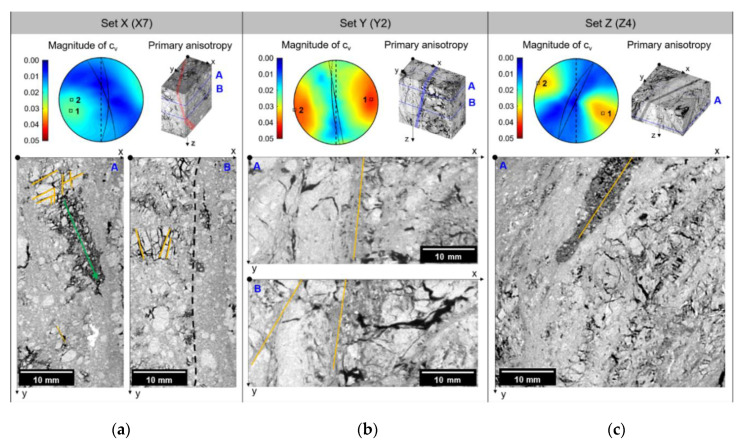
Stereonet plots of the coefficient of variation ($c_{v}$) computed by SPM and the used X-ray CT images. The orientation of the primary anisotropy is represented by a colored band in the 3D X-ray CT domain. The cross-sectional 2D images at depth A and B were obtained from the 3D X-ray CT domain. (**a**) Set X (X7). The yellow lines indicate the orientation of cracks in shattered grains. Green line shows the direction of trail suggesting the shear movements in the fault gouge. (**b**) Set Y (Y2). The yellow line shows the orientation of the narrow dark zone of matrix. This zone penetrates through the 3D domain in the z-direction. (**c**) Set Z (Z4). The yellow line indicates the orientation of the layered structure of fault gouge.

Figure 3 shows the stereonet plot of the coefficient of variation ($c_{v}$) computed by SPM. Note that the two highest values of $c_{v}$, which serve as values similar to the poles in conventional stereonet plots, are marked as square symbols, and the corresponding great circles (i.e., the anisotropic planes) are drawn as solid lines. The primary anisotropy of the stereonet, denoted as number “1”, is superimposed on a 3D X-ray CT domain. The dashed line on the stereonets indicates the great circle of the fault plane, which is parallel to the *y*-axis. The representative 2D images in the 3D X-ray CT domain are also shown and denoted by “A” and “B”.

For X7 of Set X, the magnitudes of $c_{v}$ are 0.0236 and 0.0216 for the primary and secondary anisotropies, respectively (Figure 3a). The pole of the primary anisotropy heads to 250°/15° (clockwise azimuth angle from the strike of the fault plane/elevation from the horizontal) and that of the secondary anisotropy is 270°/20°; this shows that the poles of the anisotropies are approximately subparallel to the pole of the fault plane, which corresponds to 90°/0°. Accordingly, the primary anisotropy runs along the strike of the fault plane, as illustrated by the reddish band in the 3D X-ray CT domain. The 2D image sliced at depth A shows that the local void (i.e., a very low-CT number region with dark grayscale intensity) stretches along the *y*-axis, rather than the *x*-axis. Near the *x*-axis, a grain remains shattered, and the fractures in the grain can be divided into two groups; fractures subparallel to the *x*-axis and *y*-axis, which are marked by yellowish lines. The traversing clasts, indicated by the greenish arrow, are a typical indicator of the shear displacement in the fault zone, which are known as “trails” [8]. Models for the evolution of the fabric structure or the alignment of the grains in the clay-rich gouge have been proposed for various conditions, such as saturation, the content of clay, and mineral transformation [10,14,31]. However, the fault gouge in this exhumed strike-slip fault zone includes grains with severely fractured shapes, so it is difficult to determine any preferred orientations of the grain shape. Therefore, the orientation of the particles barely contributes to its bulk anisotropy. Instead, it appears that planar discontinuities, such as fractures or trails composed of local void, affect the bulk anisotropy. The 2D image sliced at depth B exhibits two distinct regions clustered into a grain-rich zone on the left and a dark-grey zone on the right. This indicates that the average of the CT numbers decreases along the *x*-axis, and consequently the trend contributes to the anisotropy subparallel to the *y*-axis. Similar to the shattered grain in the sliced image A, the sliced image B also shows that the fault gouge contains severely shattered grains with the preserved relative position of grain fragments. This suggests that grains have been included in the fault core before they were crushed by the external stress.

Y2 of Set Y has $c_{v}$ values of 0.0420 and 0.0395 for the primary and secondary anisotropy, respectively, and the pole of primary anisotropy is 85°/10° and that of secondary anisotropy is 260°/0° (Figure 3b). The major anisotropy of Y2 is almost parallel to the fault plane. The primary anisotropy is shown as a bluish band on the 3D X-ray CT domain. The left part of the domain contrasts with the right part; the right part of the domain seems more heterogeneous compared to the opposite part. In the middle part of the 2D cross-sectional image, which is sliced at depth A, a narrow dark zone runs along the *y*-axis, which is shown as a yellowish line. The sliced image at depth B also shows narrow dark zones that run along the direction shown by the yellowish lines. The dark narrow zone penetrates the entire domain along the *z*-axis, and this constitutes the planar structure subparallel to the yz-plane, which corresponds to the fault plane. This structure contributes to the major anisotropy of Y2. Additionally, the right part of the domain is composed of a large void area and a more abundant darker matrix, compared to the left part. This contrast enhances the major anisotropy, similar to the estimated anisotropy in the stereonet.

The magnitudes of $c_{v}$ in Z4 of Set Z are 0.0353 and 0.0345 for the primary and secondary anisotropies, respectively. The pole of the primary and secondary anisotropy is directed to 115°/20° and 295°/0°, respectively. The primary anisotropy in Z4 is rotated 25° clockwise from the pole of the fault plane, shown as a black band in the 3D X-ray CT domain (Figure 3c). This also shows that a bright layer runs along the direction parallel to the black band. In the sliced image at depth A, a layered structure is found that has an orientation parallel to the strike of the great circle estimated by SPM. A dark wedge, marked as a yellowish line, is parallel to the major anisotropy and appears to be evidence of shear deformation in the same direction.

### 3.2. Anisotropy of Constituents

**Figure 4:**
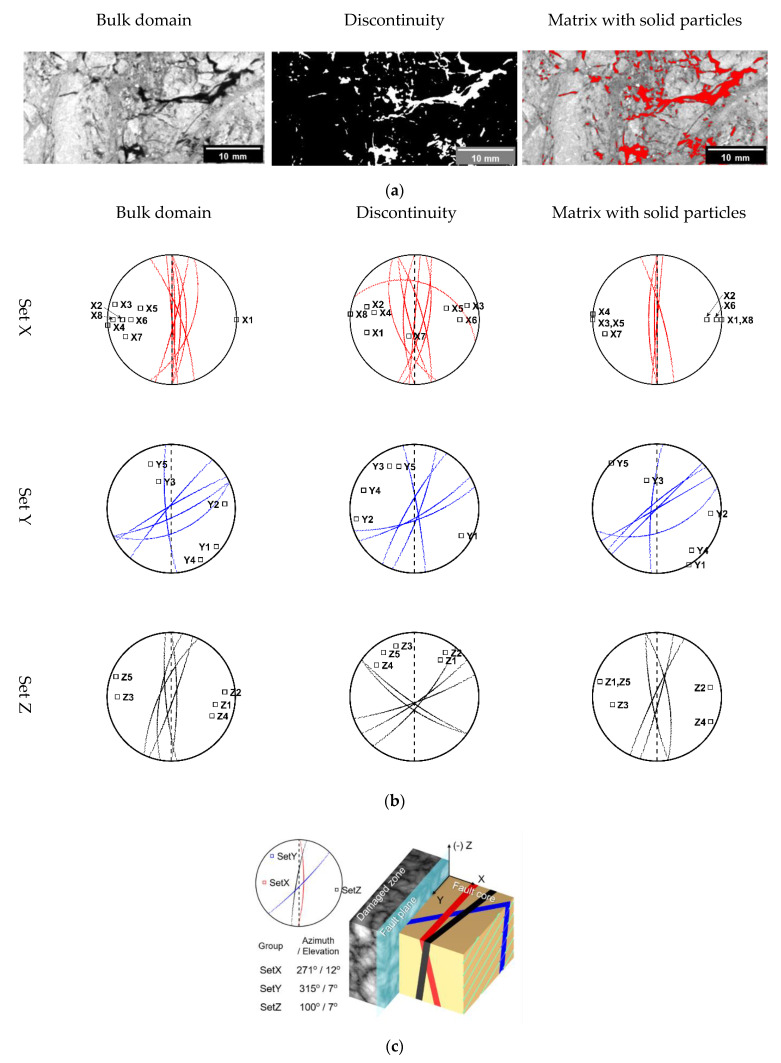
Orientation of the anisotropy of the constituents. (**a**) 2D sliced images of Y2 and the segmentation for discontinuity and the matrix with solid particles. (**b**) Primary anisotropy of the constituents for each specimen. (**c**) Average bulk anisotropy of each set.

SPM calculates the variation of the average of CT numbers along specific directions. In the previous section, SPM was implemented based on the various constituents of a bulk specimen, such as the void, matrix, and particles. Thus, it seems that the results were induced by the interplay of multiple constituents rather than a single constituent. The contrast in the CT numbers of voxels represents the difference in their phases because a CT number is proportional to the relative density of a voxel when the voxel is composed of materials with a similar effective atomic number. By separating each phase according to the CT numbers, we estimated the anisotropic orientation of the discontinuities and matrix with solid particles, respectively. Thus, the effect of the constituents on the SPM results can be verified. Firstly, the discontinuity domain was prepared as a binary structure by segmenting the low-CT number region composed of local void space (i.e., very dark gray level) from the bulk domain (Figure 4a). The voxels belonging to discontinuities have a value of 1, and the other voxel has a value of 0. We decided that the threshold for the low-CT number region was 3000 HU by visual inspection. Secondly, the matrix with solid particle domain was prepared by assigning “zero” to the discontinuity region (shown as the red area), preserving the CT numbers of the region for the matrix with solid particles. By precluding the zero region during the calculation, only the variation in the CT numbers in the matrix with a solid particle domain can be considered. Note that the range of the CT number distribution was 1000–6000 HU (Figure 2b). Thus, the preclusion of the zero region does not cause any exception in the matrix with the solid particle domain. Each primary anisotropy estimated by SPM is plotted with respect to the bulk domain, discontinuity, and matrix with solid particles (Figure 4b). The fault plane is shown as a dashed line. The magnitude of $c_{v}$ for the discontinuity domains is much higher than that of the other type of domains. This is because $c_{v}$ represents the variation in voxel values so that the contrast in voxel values in the case of the binary domain makes the magnitude of $c_{v}$ significantly higher. Thus, we focused on the orientation of the major anisotropy for each domain, instead of the magnitude of $c_{v}$.

The results of the SPM for the bulk domain show that the Set X and Set Z group have major anisotropies almost parallel to the fault plane. Also, the major anisotropies of the matrix with a solid particle domain for Set X and Set Z are largely parallel to the fault plane. On the contrary, the major anisotropies of the discontinuity domain for Set X are much more scattered than that of the other domains for Set X. Despite the discrepancy between the orientation of discontinuities and the matrix with solid particles, the results from the SPM for the bulk domain is close to the major anisotropy of the matrix with solid particles. $c_{v}$ is related to the arithmetic mean of the CT numbers so that it is governed by the volume of major clusters of CT numbers. Consequently, the magnitude of $c_{v}$ is much higher for the orientation of discontinuities because of the higher variation in voxel values, but the large volume of the matrix with solid particles determines the orientation of the bulk domain. The major anisotropies of discontinuity for Set Z are tilted from 20° to 50° in azimuth and this shows that the orientations of discontinuity of Set Z are even more scattered than that of the discontinuity of Set X. However, the major anisotropy of the matrix with solid particles of Set Z is parallel to the fault plane. Similarly, the volume of the matrix with solid particles is much larger than the volume of the discontinuity so that the major anisotropies of bulk domain for Set Z are close to the results of the matrix with the solid particle domain.

Compared to Set X and Set Z, the major anisotropies of the bulk domain for Set Y are in a much wider range, from parallel (Y2) to almost perpendicular (Y3, Y5) to the fault plane. The major anisotropies of both the discontinuity and matrix with solid particles are consistent with those of the bulk domain. In other words, the major anisotropy of bulk domains for Set Y is more scattered than that of bulk domain for Set X and Set Z, but the major anisotropy of each constituent of Set Y is more consistent. On the other hand, despite the horizontal void area in the middle of the image (Figure 4a), the major anisotropy of discontinuity for Y2 is almost parallel to the fault plane. This suggests that the void area found in the 2D image is local phenomena that does not govern the major anisotropy of the 3D image stack because the SPM finds the direction related to the maximum magnitude of $c_{v}$ through the entire volume.

The average azimuth and elevation values of the major anisotropy estimated by SPM for the bulk domain of Sets X, Y, and Z are 271°/12° for Set X, 315°/7° for Set Y, and 100°/7° for Set Z (Figure 4c). Sets X and Y have a consistent anisotropic orientation parallel to the fault plane. Set Y has a scattered orientation that presumably arises from the anomalies composed of various materials, as suggested by the dispersed CT number distribution in Figure 2b. Aside from the effect of the local anomalies, the three Sets showed that the bulk anisotropy and the anisotropy of the matrix with the solid particles are well developed along the direction subparallel to the fault plane.

Previous studies on micro-scale shear mechanisms, such as the generation of fault gouges, evolution of shear fabrics, or clay fabric orientation, were performed because macro-scale manifestations are the result of micro-scale mechanisms [5,13,32,33]. However, in this study we focused our analysis and observation on the centimeter scale for the sake of consistency between the scale of the image analysis and the experimental test. Despite the differences in the scale used in previous studies, we confirmed that shear fabrics, such as trails can be found and the bulk anisotropy of intact cataclastic rocks can be characterized using SPM. Additionally, at a centimeter scale, SPM shows that the anisotropic orientation is mainly caused by the anisotropy of the matrix with solid particles.

### 3.3. Correlation between Friction Angle and Anisotropy

**Figure 5:**
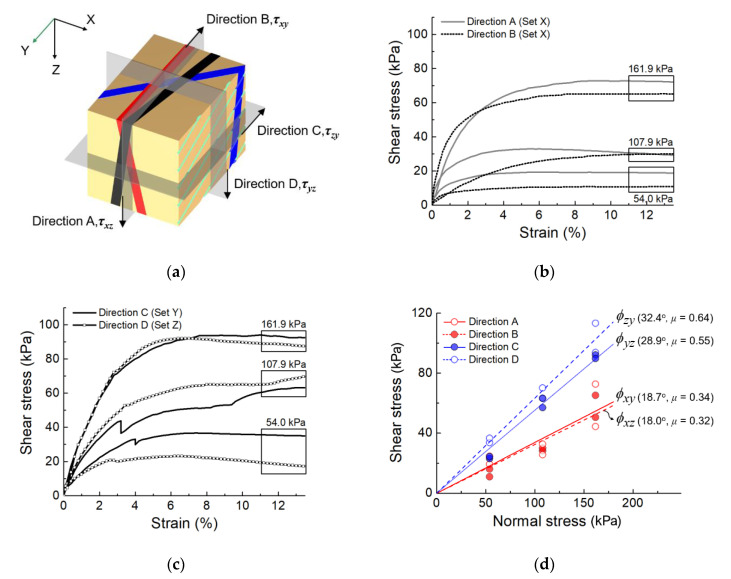
Results of the direct shear tests. (**a**) The virtual domain of the shear plane and shear direction to define τ_xy_, τ_xz_, τ_zy_ and τ_yz_. (**b**) The stress-strain curve of the Set X group sheared in Direction A and B. (**c**) Profile of the strain-stress curve obtained from the Set Y group (Direction C) and Set Z group (Direction D). (**d**) The shear strength of each group.

Figure 5a shows the shear plane and shear direction for the four tested cases; τ_xy_ and τ_xz_, were applied on the same plane parallel to the fault plane with perpendicular directions to each other. τ_zy_ and τ_yz_ were measured on the xy- and xz-planes perpendicular to the fault plane. Set X, sheared along Direction A and B (six specimens for each direction) under normal stresses of 54.0, 107.9, and 161.9 kPa, predominantly exhibits strain-hardening regardless of normal stress (Figure 5b). As the normal stress increases, the stress tends to converge towards similar asymptotic values for Direction A and B. Sets Y and Z were sheared along Direction C and D, respectively. Figure 5c shows that the shear stresses along Direction C and D are higher than those along Direction A and B. Set Y, sheared along Direction C, shows an abrupt drop in shear stress at a strain of ~3% followed by continuous evolution (the solid line in Figure 5c). At low normal stress, the evolution of stress-strain tends to be unstable in undisturbed and cohesionless soils and a larger displacement than that in the case of remolded specimens is often required for the ratio of shear stress to normal stress to converge, owing to uncertainties in natural soil [34]. As observed in the previous section, the local anomalies in Set Y, represented by the dispersion of the CT number distribution, result in unstable evolution of stress at 54 kPa and 107.9 kPa of normal stress. However, the shear stress of Direction C approaches that of Direction D at large strain, particularly for normal stress of 162 kPa, under which instability of stress was not observed.

The maximum shear strength was taken at 10% of strain unless the peak stress was clearly identified, and the Coulomb failure criteria were applied to compute the friction angle with an assumption of non-cohesion. Conventionally, a fault gouge is classified as incohesive cataclastic rock in a depth less than 4 km [35]. Along Direction A and B, the friction angle values were estimated to be over 18.0° (corresponding to *μ* = 0.32), and the friction angle values along Direction C and D were estimated to be approximately 30° (corresponding to *μ* = 0.57). This suggests that the fault gouge has low frictional resistance when the shearing plane coincides with the embedded anisotropic structure. This experimental result is corroborated by previous observations that the coincidence of weak constituents and the fabric structures accounts for the low shear strength of the fault core [36,37,38,39].

## 4. Discussion

In this paper, we characterized the bulk anisotropy in fault gouge specimens based on 3D μ-CT images and the correlation between the estimated anisotropy and experimentally measured friction angles. A total of 24 undisturbed specimens were cored in three orthogonal directions and subjected to SPM and direct shear test. The statistical analysis using SPM showed that the major orientation of anisotropy is a result of the spatial distribution of all constituents. The frictional resistance measured along different shearing directions highlights the contribution of the anisotropic structure to low shear resistance in fault gouges. The results are summarized as follows. The systematic evaluation of CT numbers by SPM shows that, regardless of coring direction, the tested specimens have clear anisotropic structures subparallel to the fault plane at core-scale. The discontinuity, layered bands, void, and fractures contribute to the anisotropy, all of which were inferred via visual observation using 2D sliced and 3D μ-CT images for Sets X, Y, and Z. Image-based analysis has the advantage of allowing the quantitative identification of the anisotropy when the orientation of in situ samples is unknown. The lowest shear resistance is obtained when the anisotropic structure is parallel to the shear plane, regardless of shear direction. However, the shear strength tends to increase when shearing occurs perpendicular to the anisotropy in the fault core, where it exhibits an approximately 178% higher frictional coefficient than in the other cases. This experimental study shows that quantification of the anisotropy of all the constituents in undistributed fault core specimens is correlated with the anisotropy of the shear resistance even at core-scale.

Previous studies have investigated fault gouges using remolded and synthetic specimens to examine either the constitutive parameters or frictional coefficients under normal stress ranging over tens of MPa [10,36,38,40,41]. The direct shear test in this study was conducted under normal stresses of up to hundreds of kPa to preserve the actual structure of the undisturbed fault gouge. Despite the difference in the range of normal stress, the discrepancy in the frictional coefficients resulting from the different shearing direction is in good agreement with the discrepancy in the frictional coefficients of the Zuccale fault owing to the difference in the sample preparation method (Table 1). The frictional coefficients were obtained using two types of specimens: one was a natural undisturbed specimen and the other was natural but remolded to ignore the effect of the fabric structures [37]. Also, it was confirmed that the overall range of the frictional coefficients corresponds to the range found in the literature. Although this study did not investigate the mineralogical composition, the estimation of 3D structural anisotropy based on μ-CT images provides some insight into the microstructure of fault gouges. This study confirmed that the layered structure of the matrix with a solid particle domain majorly contributes to the bulk anisotropy of a fault gouge. The relationship between a CT number, the relative density, and the effective atomic number of the materials states that the layered structure of the matrix with a solid particle domain is caused by (1) the anisotropy of structure that results from the differences in the relative density of materials, or (2) the anisotropy of minerals that result from the differences in the effective atomic number. In the previous study, the variation in CT numbers in X-ray CT images of fault gouges mainly results from the relative density of the materials as observed using optical microscopy and SEM-EDS [21]. It showed that the comparably low CT number was induced by the network of open cracks inside the voxel due to the dilative shear deformation. This suggests that the results estimated by SPM for the X-ray CT image of fault gouges are highly correlated with the microstructure of nested cracks.

On the other hand, the layered structure subparallel to the fault plane observed in this study can be examined further by using a conceptual model for wide strike-slip fault zones represented as a layered structure composed of phyllosilicate-rich gouges and lenses of severely fractured protolith [7]. The findings of this study are consistent with those of previous studies, further highlighting the feasibility of image-based analyses to quantitatively understand the microstructural features of gouges.

## 5. Conclusions

This study presents the application of statistical analysis, SPM, to the quantification of 3D structural anisotropy embedded in undisturbed natural fault gouges by using 3D images obtained from X-ray CT scanning. Also, the meaning of the structural anisotropy estimated by SPM was verified with the anisotropy of frictional resistance measured by direct shear tests conducted on the same specimens used in the X-ray CT scanning. The following conclusions can be drawn.

- The undisturbed specimens were cored along three orthogonal directions, and were subjected to X-ray CT scanning followed by SPM. The results show that despite the varied coring directions, the SPM provides consistent evaluations of the 3D structural anisotropy of the fault gouges. It was confirmed that the core-scale orientation of the structural anisotropy is almost parallel to the fault plane, and the main contributor to the anisotropy is the layered structure of the matrix with solid particles in natural fault gouges.
- After the X-ray CT scanning, direct shear tests were conducted under different shearing directions and shear planes. The frictional coefficient is lowest when the shearing occurs on the shear plane parallel to the fault plane, and the frictional coefficient doubles when the shear plane is perpendicular to the fault plane.
- The results show that the structural anisotropy identified by SPM and the anisotropy of frictional resistance of fault gouges are correlated to the orientation of fault plane. This suggests that the evaluation of structural anisotropy by SPM indicates the weakening direction of the frictional resistance of fault gouges.

## Figures and Tables

**Table 1:** Frictional strength of foliated gouges as measured in previous studies.

References	Frictional Coefficients at Steady State	Specimen Type	Normal Stress Range	Location
Collettini et al. (2009)	0.25–0.31	Intact	10–150 [MPa]	Zuccale fault, Italy
Collettini et al. (2011)	0.55–0.62	Powdered	10–150 [MPa]	Zuccale fault, Italy
Lockner et al. (2011)	0.15	Powdered	40–200 [MPa]	San Andreas fault, United States
Kato and Hirono (2016)	0.2–0.4	Powdered	0.5–2.5 [MPa]	Atera fault, Japan
Numelin et al. (2007)	0.3–0.4	Remolded	5–150 [MPa]	Panamint Valley fault zone, United States
